# Supplementary material for: Computational analysis of GAL pathway pinpoints mechanisms underlying natural variation
Source: PLoS Comput Biol. 2021 Sep 27;17(9):e1008691. doi: 10.1371/journal.pcbi.1008691 (PMC8496860; doi:10.1371/journal.pcbi.1008691)
Supplement: S1 Table — (DOCX) [file pcbi.1008691.s011.docx]

| Natural isolate | Genotype | Lineage | Ecological niche |
| --- | --- | --- | --- |
| 273614N | MATa/x; hoΔ::GAL1pr-YFP-hphNT1 | Wine/European | Clinical isolate |
| BC187 | MATa/x; hoΔ::GAL1pr-YFP-kanMX4 | Wine/European | Barrel fermentation |
| CLIB324 | MATa/x; hoΔ::GAL1pr-YFP-hphNT1 | Mosaic | Baker strain |
| DBVPG1853 | MATa/x; hoΔ::GAL1pr-YFP-hphNT1 | Mosaic | White Tecc |
| DBVPG6765 | MATa/x; hoΔ::GAL1pr-YFP-hphNT1 | Wine/European | Lici fruit |
| I14 | MATa/x; hoΔ::GAL1pr-YFP-hphNT1 | Mosaic | Soil |
| IL-01 | MATa/x; hoΔ::GAL1pr-YFP-hphNT1 | Mosaic | Soil |
| L-1528 | MATa/x; hoΔ::GAL1pr-YFP-hphNT1 | Wine/European | Fermentation |
| S288C | MATa/x; hoΔ::GAL1pr-YFP-hphNT1 | Mosaic | Rotting fig |
| UWOPS87-2421 | MATa/x; hoΔ::GAL1pr-YFP-hphNT1 | Mosaic | Cladode, Opuntia megacantha |
| Y12-SGRP | MATa/x; hoΔ::GAL1pr-YFP-kanMX4 | Sake | Palm wine strain |
| YJM975 | MATa/x; hoΔ::GAL1pr-YFP-hphNT1 | Wine/European | Clinical isolate |
| YJM981 | MATa/x; hoΔ::GAL1pr-YFP-hphNT1 | Wine/European | Clinical isolate |
| YPS163 | MATa/x; hoΔ::GAL1pr-YFP-hphNT1 | North American | Oak tree |
| YPS606 | MATa/x; hoΔ::GAL1pr-YFP-hphNT1 | North American | Oak tree |

**S1 Table: List of strains used in this study.**
