# Supplementary material for: Computational analysis of GAL pathway pinpoints mechanisms underlying natural variation
Source: PLoS Comput Biol. 2021 Sep 27;17(9):e1008691. doi: 10.1371/journal.pcbi.1008691 (PMC8496860; doi:10.1371/journal.pcbi.1008691)
Supplement: S2 Table — (DOCX) [file pcbi.1008691.s012.docx]

| Parameter | Description | Value | Units |
| --- | --- | --- | --- |
| a1 | Basal Gal1p production rate | 0.001 | nM/min |
| a2 | Basal Gal2p production rate | 0.020 | nM/min |
| a3 | Basal Gal3p production rate | 0.164 | nM/min |
| a4 | Basal Gal4p production rate | 0.063 | nM/min |
| a80 | Basal Gal80p production rate | 0.394 | nM/min |
| aR | Mig1p production rate | 0.770 | nM/min |
| ag1 | Maximum Gal1p production rate | 627.644 | nM/min |
| ag2 | Maximum Gal2p production rate | 6 | nM/min |
| ag3 | Maximum Gal3p production rate | 2.525 | nM/min |
| ag4 | Maximum Gal4p production rate | 0.007 | nM/min |
| ag80 | Maximum Gal80p production rate | 0.389 | nM/min |
| a0HXT | Basal HXT production rate | 0.060 | nM/min |
| aHXT | Maximum HXT production rate | 11.320 | nM/min |
| d | Degradation rate of proteins | 0.009 | 1/min |
| dsugar | Turnover rate of sugar | 7 | 1/min |
| kf3 | Forward binding rate of galactose to Gal3p | 75.160 | 1/(nM·min) |
| kr3 | Unbinding rate of galactose from Gal3p | 3391 | 1/min |
| kf83 | Forward binding rate of Gal3p to Gal80p | 11222 | 1/(nM·min) |
| kr83 | Unbinding rate of Gal3p from Gal80p | 700 | 1/min |
| kf84 | Forward binding rate of Gal80p to Gal4p | 596 | 1/(nM·min) |
| kr84 | Unbinding rate of Gal80p from Gal4p | 1237 | 1/min |
| KG1 | GAL1 transcriptional threshold for Gal4p activation | 16.820 | nM |
| KG2 | GAL2 transcriptional threshold for Gal4p activation | 23.510 | nM |
| KG3 | GAL3 transcriptional threshold for Gal4p activation | 7.500 | nM |
| KG80 | GAL80 transcriptional threshold for Gal4p activation | 12.920 | nM |
| KR1 | GAL1 transcriptional threshold for Mig1p inhibition | 23.100 | nM |
| KR3 | GAL3 transcriptional threshold for Mig1p inhibition | 1004 | nM |
| KR4 | GAL4 transcriptional threshold for Mig1p inhibition | 26.230 | nM |
| KRs | Equilibrium constant of glucose binding to Mig1p | 8.360 | nM |
| KHXT | HXT transcriptional threshold for Gal4p inhibition | 5.090 | nM |
| kG2 | transportation rate through Gal2p | 1.200 | 1/min |
| rcat | the ratio of transportation rate through HXT and through Gal2p | 1.8 | Dimensionless |
| rG2 | the ratio of galactose and glucose binding affinity to Gal2p | 0.45 | Dimensionless |
| rHXT | the ratio of galactose and glucose binding affinity to HXT | 100 | Dimensionless |
| KGglu | Equilibrium constant of glucose binding to Gal2p | 36000000 | nM |
| KHXTglu | Equilibrium constant of glucose binding to HXT | 540000 | nM |
| n1 | GAL1 Hill coefficient for Gal4p activation | 3 | Dimensionless |
| n2 | GAL2 Hill coefficient for Gal4p activation | 2 | Dimensionless |
| n3 | GAL3 Hill coefficient for Gal4p activation | 2 | Dimensionless |
| n80 | GAL80 Hill coefficient for Gal4p activation | 2 | Dimensionless |
| nR1 | GAL1 Hill coefficient for Mig1p inhibition | 2 | Dimensionless |
| nR3 | GAL3 Hill coefficient for Mig1p inhibition | 1 | Dimensionless |
| nR4 | GAL4 Hill coefficient for Mig1p inhibition | 1 | Dimensionless |
| nRs | Mig1p Hill coefficient for glucose activation | 2 | Dimensionless |
| nHXT | HXT Hill coefficient for Gal4p inhibition | 10 | Dimensionless |

**S2 Table: Parameter descriptions, units, and ‘best-fit’ values.**
