# Supplementary material for: Computational analysis of GAL pathway pinpoints mechanisms underlying natural variation
Source: PLoS Comput Biol. 2021 Sep 27;17(9):e1008691. doi: 10.1371/journal.pcbi.1008691 (PMC8496860; doi:10.1371/journal.pcbi.1008691)
Supplement: Attachment — Submitted filename: Response to the reviewers.docx [file pcbi.1008691.s013.docx]

Response to the reviewers’ comments

Reviewer #1:

*Many genetic studies focus on unraveling the different alleles that contribute to a quantitative trait. Such QTL or GWAS studies typically only identify a specific genomic region, and thus require more experimental work to pinpoint the exact alleles that dive the observed phenotypic variation. Moreover, in many cases, the underlying molecular mechanism (i.e. how a given genetic variation might influence a quantitative trait) remains obscure. This study by Hong and coworkers shows how mathematical models can help to predict the possible molecular mechanisms underlying natural variation in a nutrient-sensing network. Such models could be a formidable tool to help predict and understand if and how specific genetic variation (alleles) might influence complex, quantitative phenotypes.*

*Specifically, the authors describe the development of a model that recapitulates the complex behavior of the galactose network in the eukaryotic model S. cerevisiae. The authors previously used a clever setup to characterize the dynamic, quantitative behavior (input-output response) of the GAL network and found that in general, yeast cells activate the GAL genes when the ratio of the concentration of galactose to glucose reaches a certain threshold. Around the threshold, the system is bimodal and unstable, resulting in variability within an isogenic population. Moreover, the exact sugar ratio required to activate or inactivate the network, as well as the bimodality and induction levels vary between different S. cerevisiae genetic backgrounds, making the GAL response a good model for a complex, quantitative trait. In this new work, using a model that recapitulates the GAL network, the authors show that the variation between the GAL response of natural yeast strains can be explained by changes in a few relatively simple parameters, such as the ration of the glucose and galactose binding affinity for the shared transporter Hxt, the binding rate between Gal3 and Gal80, the binding rate between galactose and Gal80 or the synthesis rate of Mig1. Hence, the model predicts that mutations/alleles that would affect these parameters could explain the observed natural variation in the GAL phenotypes.*

*general assessment:*

*Overall, the work is interesting to the broad range of colleagues interested in QTL analyses as well as those modeling regulatory pathways. The text is exceptionally well-written and the work seems solid. As such, I think it is perfectly suited for PLoS Comp Biol.*

We thank the reviewer for careful reading of our manuscript and for the comments which we addressed as detailed below.

(1) *Major comment*

*My only concern is that the authors fail to validate their predictions. Ideally, the authors would complement this study with a QTL analysis (eg pooled segregant analysis) to pinpoint some of the alleles underlying the observed variation in some of the natural yeast strains, and then relate this back to their model, to show that the model indeed recapitulated reality. I realize that this is a lot to ask and goes beyond the scope of the paper. So instead, it might be possible to come up with some more simple validation tests, eg by changing Mig1 expression or mutating Gal80, investigate the effect of these genetic perturbations on the GAL behavior and discuss whether this is in line with what would be expected from the model? Or perhaps the authors have another idea how they could validate their predictions?*

We thank the reviewer for the suggested experiment. To test our predictions, we introduced the GAL3 allele from YPS606 into a S288C*^gal3Δ^* genetic background, then measured the induction profiles in the resulting engineered strain. As predicted, we showed that the decision threshold shifted toward a higher glucose/galactose ratio. This data is included as a new figure panel (Figure 5h).

(2) *Minor: Line 102 could use a reference.*

We have added the reference.

Reviewer #2:

*In this manuscript, Hong et al. use an ODE model of the GAL pathway in yeast to try to explain variation in bimodality in among natural isolates. The Springer lab has been using the GAL pathway to explore the mechanistic basis of quantitative genetic variation, and this paper extends that work. They examined 15 natural isolates and found variation in the ratio of glucose to galactose at which the GAL pathway is induced as well as in the induction levels of the pathway. They built a somewhat simplified ODE model of the pathway (similar to other models in the literature) and fit it to match the induction behavior of a standard lab strain and two mutants with deletions of key genes in the pathway. Starting from this parameter fit, they systematically perturbed parameters to see if they could identify ones that affected the bimodal range and then to find ones which, when perturbed, produced a better match to the experimental data for the natural isolates.*

*In general this study, as presented, follows a logical progression. Natural variation is observed; a generative model is produced and fit to a reference strain(s); parameters of the model are perturbed to see if they can explain the natural variation.*

*The experimental data seems solid. The Springer lab has published these kinds of experiments for a while now and the new thing here is that they use new strains.*

*The model is a variant of commonly used ODE models of the GAL pathway. They include a few more details than other models about sugar uptake and intracellular sugar dynamics, which are useful additions, especially given the importance of glucose:galactose ratios in inducing the pathway.*

We thank the reviewer for careful reading of our manuscript and for the comments. We have addressed the issues as detailed below.

(1) *One thing that isn't clear to me is whether the results shown in figure 2b are good fits to the experimental data. There are substantial quantitative differences in the GAL induction levels for all three strains between the simulations and experiments. This is not terrible - the model is a simplification after all - but the authors should discuss it and discuss the implications of this less than ideal fit for the investigations that follow. They used an objective function that summarized the differences between this heat maps but there isn't any insight given into how far off the simulations were and what the landscape looks like.*

We have added the sentences below in main text and made a clearer reference to Fig S1 which also shows how far off the simulations were away from experimental data.

“Although there are quantitative differences in the GAL induction levels for all three strains between the simulations and experiments (*Obj^WT^* = 7.59, *Obj^mig1Δ^* = 7.80, *Obj^gal80Δ^* = 14.88), and the overall fitting was not as good as only fitting to wildtype S288C data (as shown in Fig S1, *Obj* = 6.76), this parameter set captured the ratiometric response in the wildtype S288C, the elevated induction levels in S288C*^mig1Δ^*, and the glucose threshold sensing in S288C*^gal80Δ^* (*GAL1* induction levels only respond to glucose titration and are insensitive to changes in galactose concentration, as data of S288C*^gal80Δ^* showing in Fig 2b). Meanwhile, simultaneously fitting to three strains data avoids overfitting to a specific strain and retains the generality to recapitulate common characteristics shared by natural yeast isolates. Hence, we stuck with this parameter set as starting parameters in our following investigations.”

(2) *It's also important to note that for some of the parameters (and some key parameters like rHXT and ag2) the wildtype parameter distribution spanned at least 1 and sometimes close to 2 orders of magnitude. Later, when they explore the effect of parameter value perturbations, they do it over 4 orders of magnitude (2 up, 2 down). Does it matter that these particular parameters are not very well constrained?*

The fact that some parameters can vary by 1 to 2 orders of magnitudes just highlights that these exact parameter values do not strongly influence the systems level behaviour. In other cases, the parameter can be important but there can be a broad range over which the parameter values can change before having an effect on the system.

(3) *Figures S2-S5 show the effects of changing various parameters on the deltaON, deltaFULL, and deltaLEVEL metrics. These figures are hard to read. There is a lot going on, and I'm not sure how to fix them. Perhaps splitting the ON,FULL,lEVEL into their own groups instead of having them side by side within each glucose concentration might help.*

We used all the three metrics at 8 glucose concentrations as input for subsequent clustering, thus we retain Fig S2 to show the comprehensive information. In addition, we used Fig S3-S5 to show the effect of parameter perturbations in each cluster. With that said, we understand that Figure S2-S5 contain a considerable amount of information, so we added Fig S6 to show the effect of parameter perturbations on individual metrics, as suggested by the reviewer.

(4) *In Figure 4, they show how changing various parameters improves the fit of the model. This is based on equation 2. What does "min" in this equation mean? What is the minimization taken over? On that note, for these natural isolate fits, are the fits just tested at the 2-fold, 10-fold, 100-fold levels or are they allowed to slide continuously, one parameter at a time?*

We have added the following explanation in Materials and Methods section to clarify. “To generate Fig 4, the parameters were scanned across 6 orders of magnitude centered on the starting parameter (3 up, 3 down), one parameter at a time. For each parameter, 48 points were evenly sampled in logarithmic space across these 6 orders of magnitude flanking the default parameter value. The minimization was taken over the resultant objective function.”

(5) *In line 292, the authors say they simulated the induction profile for 8 strains. How does this differ from the assessment they did in the previous paragraph? Didn't they already have the simulation results? Why would they need to simulate them again? If I am misunderstanding the steps and the difference between the two parts, please make them a little more clear.*

We used the simulation results in Fig 4 to visualize the induction profiles shown in Fig 5, there were no extra simulations. We have changed the wording as follows to avoid confusion.

“We subsequently used heatmap to visualize the induction profiles of the 7 strains that showed a fitting improvement ≥70% in Fig 4.”

(6) *As with figure 2, for figure 5 I can look at the simulated vs. experimental heat maps and accept that they look similar in some ways and fir some strains more than others, but there are clear differences and perhaps changing a different parameter would also make a heat map that looked acceptably close. It would be nice to see a graph with a quantification of how much better changing this parameter did than changing the others and how close it got to the actual data.*

Figure 4 shows a comprehensive quantification of how much better changing one parameter is compared to changing the others. To give an intuitive sense of the difference between tuning different parameters, we have added Fig S8 to show the comparison and how closely it matched the actual data.

(7) *This leads to my final point. The authors have used the model to make a clear hypothesis for what is causing the differences between the strains, or at least the ones in figure 5. But at this point, it's just a hypothesis, and we don't really know whether this whole exercise was useful in explaining the actual difference between natural isolates or just an exercise in exploring the behavior of the model. It would be nice to have some experimental verification. This isn't trivial, especially for the rHXT parameter since this is a composite of several genes, but perhaps YJM981 and YPS606 could be tested with allele swaps. For YPS606 they did this allele swap in a previous paper (at least in one direction). It isn't clear to me whether they did the full heat map, but they presumably already have the strain. For YJM981, perhaps a MIG1 promoter swap would be appropriate. It would be even better if they could figure out a way to swap the Gal80-Gal3 binidng interfaces so as to affect the kf83 parameter because then they could test three of their strains. Again, it isn't necessarily straightforward to do these (except perhaps for YPS606), but it would provide an unassailable validation of their approach.*

We thank the reviewer for the suggested experiment. To test our predictions, we introduced the GAL3 allele from YPS606 into a S288C*^gal3Δ^* genetic background, then measured the induction profiles in the resulting engineered strain. As predicted, we showed that the decision threshold shifted toward a higher glucose/galactose ratio. This data is included as a new figure panel (Figure 5h).

(8) *Minor note: line 678 - "Besides" is the wrong word.*

*line 368 - "simply" is the wrong word.*

We have changed the wording.

Reviewer #3:

*In this work, the authors developed an ODE model of the essential parts of the GAL metabolic network in s. cerevisiae that captures the experimental response of natural isolates of yeasts to combinations of glucose and galactose.*

*The authors performed fit of the model to WT, gal80d, and mig1d and show that, besides the parameters that are associated with the delete, the same parameters capture induction levels of all strains.*

*The authors then perform a parameters scan and quantify the changes in three parameters that are relevant to the bimodal nature of the system and identify a few typical classes of perturbation. The authors also quantify the difference in the parameters the fit the data of the different natural strains. Finally, the author demonstrates how a change of single parameters can capture the changes in the decision fronts.*

*The goal to connect macroscopic phenotypic changes to changes in microscopic parameters is important and interesting. The GAL system provides a simple yet rich system to demonstrate the value of connecting microscopic models to macroscopic phenomena.*

We thank the reviewer for careful reading of our manuscript and for the comments. We have addressed the issues as detailed below.

*I have a few minor comments:*

1) *Decision front: It would be beneficial to explain more about why the decision fronts are straight lines (in the log-log space) and more details on how they are calculated (half maximum of the whole plate? of isoglucose line?) and why they span differently in different strains.*

We included the following discussion of the decision threshold in the main text for clarity.

“The decision thresholds are calculated as half maximum induction of the GAL pathway (compared to its maximal expression in the 96 different conditions in which the isolates were queried). As the decision threshold depends on both glucose and galactose, each isolate has many “decision thresholds”. These decision thresholds are linked together by the red lines in Fig 1b; we term this curve the “decision front”. The decision fronts are essentially contour lines that join nutrient conditions which give rise to equal induction levels of the GAL pathway. Galactose activates the pathway via a positive feedback, whereas glucose inhibits the pathway through carbon catabolite repression. As a result, the induction level is approximately proportional to galactose concentration, while inversely proportional to glucose concentration. This leads to the decision front appearing as a straight contour line in log-log space. As different strains have various sensitivities to galactose induction and glucose inhibition, each strain has a characteristic decision front, representing the concentration ratio required to induce the GAL network in that strain.”

2) *Add a plot of the phase space that will allow estimating the hysteresis of the system.*

Fig 3a depicts hysteresis from varying external galactose. The exact induction level depends on specific parameters that are used to simulate it. We have attached here an example of the simulation with original parameters and perturbed kf3.

3) *When analyzing the bimodality, it would be interesting to add the natural strain distribution on the tSNE to see the natural variation span with respect to the clusters. Along the same lines, it would be insightful to perform some non-linear embedding analysis (e.g., diffusion map) to the natural stains data.*

We appreciate the interesting idea proposed by the reviewer, the problem to superimpose natural strains on top of the tSNE projection is that in Fig 3b, we are clustering parameter perturbations according to their effect on GAL induction, thus each data point in the plot corresponds to a specific perturbation, instead of a simulated strain. We tried to perform non-linear embedding analysis to the natural strains data, attached here is a tSNE projection based on induction profiles of natural strains. We suspect that the lack of a striking result arises in part from the limited number of data points.

4) *It would be insightful to include a figure that compares the region of bimodality with the best fit.*

Figure S1 compares the region of bimodality with the best fit. We reference this in the text as follows:

“To identify regions where bistability (and hence bimodality) exists, we started from either high galactose or no galactose initial conditions and allowed the system to evolve towards steady state. These simulations recapitulated the three basic responses we saw experimentally: 1) an area where all cells repressed the GAL pathway (unimodal OFF), 2) an area where all cells induced the GAL pathway (unimodal ON), and 3) a mixed population with some cells repressing and some cells inducing the pathway (bimodal) (Fig S1).”

5) *Including representative examples for the actual dynamics of each cluster of 3b would help to understand the change at each cluster.*

We have added Figure S7 to include representative examples of each cluster in Figure 3b.

6) *what is the impact of the single parameter changes that explain the decision front movement (fig. 5) on the bimodality parameters? is the change consistent?*

We thank the reviewer for this question. We went back and checked the bimodality parameters for all experiments. The bimodality parameters were consistent with the decision front movement shown in Fig 5 (decreasing rHXT, increasing kf83, and increasing kf3 all lead to GAL induction at lower galactose concentration) except in one case. We have eliminated the panel (old Fig 5h) from the paper (parameter scan of aR). We attached here the simulation results for rHXT and kf3 for reference, we have also added the simulation result for kf83 in Figure S7.

7) *The first sentences in the abstract that mention human traits seem detached from the rest of the paper and should be removed.*

We have modified the abstract, specifically we removed clauses regarding human height and human genetic diseases.
